# Causal Associations Between Educational Attainment and 14 Urological and Reproductive Health Outcomes: A Mendelian Randomization Study

**DOI:** 10.3389/fpubh.2021.742952

**Published:** 2021-10-28

**Authors:** Menghua Wang, Zhongyu Jian, Xiaoshuai Gao, Chi Yuan, Xi Jin, Hong Li, Kunjie Wang

**Affiliations:** ^1^Department of Urology, Institute of Urology (Laboratory of Reconstructive Urology), West China Hospital, Sichuan University, Chengdu, China; ^2^West China Biomedical Big Data Center, Sichuan University, Chengdu, China

**Keywords:** educational attainment, Mendelian randomization, urology, reproductive health, oncology

## Abstract

**Background:** The impact of educational attainment (EA) on multiple urological and reproductive health outcomes has been explored in observational studies. Here we used Mendelian randomization (MR) to investigate whether EA has causal effects on 14 urological and reproductive health outcomes.

**Methods:** We obtained summary statistics for EA and 14 urological and reproductive health outcomes from genome-wide association studies (GWAS). MR analyses were applied to explore the potential causal association between EA and them. Inverse variance weighted was the primary analytical method.

**Results:** Genetically predicted one standard deviation (SD) increase in EA was causally associated with a higher risk of prostate cancer [odds ratio (OR) 1.14, 95% confidence interval (CI) 1.05–1.25, *P* = 0.003] and a reduced risk of kidney stone (OR 0.73, 95% CI 0.62–0.87, *P* < 0.001) and cystitis (OR 0.76, 95% CI 0.67–0.86, *P* < 0.001) after Bonferroni correction. EA was also suggestively correlated with a lower risk of prostatitis (OR 0.76, 95% CI 0.59–0.98, *P* = 0.037) and incontinence (OR 0.64, 95% CI 0.47–0.87, *P* = 0.004). For the bioavailable testosterone levels and infertility, sex-specific associations were observed, with genetically determined increased EA being related to higher levels of testosterone in men (β 0.07, 95% CI 0.04–0.10, *P* < 0.001), lower levels of testosterone in women (β −0.13, 95% CI−0.16 to−0.11, *P* < 0.001), and a lower risk of infertility in women (OR 0.74, 95% CI 0.64–0.86, *P* < 0.001) but was not related to male infertility (OR 0.79, 95% CI 0.52–1.20, *P* = 0.269) after Bonferroni correction. For bladder cancer, kidney cancer, testicular cancer, benign prostatic hyperplasia, and erectile dysfunction, no causal effects were observed.

**Conclusions:** EA plays a vital role in urological diseases, especially in non-oncological outcomes and reproductive health. These findings should be verified in further studies when GWAS data are sufficient.

## Introduction

It is well-established that educational attainment (EA) is an essential social determinant of health (1). A prior study reported that EA was correlated with many health outcomes, including adiposity, diabetes, and coronary artery diseases (2), suggesting the non-negligible role of EA in health.

In the field of urology and reproductive medicine, there have also been some observational studies that investigated the correlation between EA and health outcomes, namely, prostate cancer (3, 4), bladder cancer (5), kidney cancer (6), testicular cancer (7), kidney stone (8, 9), benign prostatic hyperplasia (BPH) (10, 11), prostatitis, cystitis, incontinence (12, 13), erectile dysfunction (ED) (14), male infertility (15), female infertility (15–17), and testosterone levels among males and females (18), showing that EA might play a vital role in urological and reproductive health. However, there are few relevant studies, and the results from prior studies were partially inconsistent. Additionally, existing observational studies are vulnerable to confounding factors and reverse causality.

Mendelian randomization (MR) is a genetic epidemiological method that applies genetic variants, such as single nucleotide polymorphisms (SNPs), to estimate the causal effect of an exposure (e.g., EA) on an outcome (e.g., kidney stone). Compared with conventional observational studies, this method is less vulnerable to confounding factors and reverse causation and has been widely used in current epidemiological studies (19).

Recently, a large-scale genome-wide association study (GWAS) identified genetic variants associated with EA (20), which provides high-quality genetic instruments for us to estimate the causal effects of EA on health outcomes. The genetic variants derived from this GWAS have already been used to evaluate the causal effects of EA on osteoarthritis (21) and diabetes (22).

As a result, in the current research, we used MR analysis to determine the causal effect of EA on the 14 urological and reproductive health outcomes mentioned above, to provide new insights into the role of EA in these health outcomes.

## Materials and Methods

We performed the current MR study based on the Strengthening the Reporting of Observational Studies in Epidemiology (STROBE) guideline (Supplementary Table 1). The overall study design of the current MR analysis is presented in Supplementary Figure 1.

### Instrumental Variables Selection

We used SNPs that were identified to be correlated with EA from a GWAS performed by the Social Science Genetic Association Consortium (20). This GWAS was a meta-analysis of 71 cohort-level studies that enrolled 1,131,881 individuals of European ancestry. Education attainment was measured as the number of years of schooling that participants completed. Although there are differences in education systems for EA between cohorts, the International Standard Classification of Education system was applied to match education qualifications across the cohorts. Under the threshold of *P* < 5 × 10^−8^ and pairwise *r*^2^ < 0.1, the GWAS identified 1,271 SNPs that are correlated with EA, which explained 11–13% of the variance. Among the 1,271 SNPs, the SNPs with potential linkage disequilibrium (pairwise *r*^2^ > 0.01), those not found in the GWAS outcome datasets, and those that were palindromic with intermediate allele frequencies were excluded. Since the quality of the instrumental variables was essential for the MR study, we used the *F* statistics to evaluate the strength of the instrumental variables. Although we did not calculate the *F* statistics specifically in the current study, a prior study that investigated the association between EA and osteoarthritis using similar SNPs as our study reported a median *F* statistics of 45 (21), suggesting that the instrument strength was generally reliable. The SNP coefficients were per standard deviation (SD) units of years of schooling (SD = 4.2 years).

### GWAS Data Sources for 14 Urological and Reproductive Outcomes

We extracted summary statistics for prostate cancer (79,148 cases and 61,106 controls) from the Prostate Cancer Association Group to Investigate Cancer-Associated Alterations in the Genome (PRACTICAL) Consortium (23). The genetic variants for bioavailable testosterone levels were extracted from a gender-specific GWAS performed in the UK Biobank (178,782 men and 188,507 women) (24). The UK Biobank is a large-scale biomedical database and has been widely used in the field of health. Summary statistics for ED were obtained from another GWAS with 6,175 cases and 217,630 controls in total (25). We obtained the genetic variants for the remaining 10 outcomes, including bladder cancer (1,115 cases and 174,006 controls), kidney cancer (971 cases and 174,006 controls), testicular cancer (199 cases and 74,685 controls), kidney stone (4,969 cases and 213,445 controls), BPH (13,118 cases and 72,799 controls), prostatitis (1,859 cases and 72,799 controls), cystitis (8,081 cases and 195,140 controls), incontinence (1,357 cases and 202,910 controls), male (680 cases and 72,799 controls), and female (6,481 cases and 68,969 controls) infertility from the latest R5 release of the FinnGen project. The FinnGen project is an ongoing project combining the genotype data and digital health record data of Finnish individuals, which provides a high-quality database for researchers to explore genetic variation in diseases. Detailed information about the FinnGen project can be found at their official site (26). A description of the 14 urological and reproductive health outcomes, including data sources, sample size, and definitions, is presented in Supplementary Table 2.

### Statistical Analysis

Inverse variance weighted (IVW) was the primary analytical method in our study, which could provide the most precise causal estimates (27). Additionally, we performed several sensitivity analyses to validate our findings, including MR-Egger, weighted median, and weighted mode. MR-Egger is a method that can provide estimates after the correction of pleiotropy (28). The weighted median method could generate reliable estimates even if up to 50% of weights come from invalid instruments (29). The weighted mode has natural robustness to outlying variants (30). We used the MR-Egger intercept to examine directional pleiotropy and Cochrane's *Q-*test to estimate heterogeneity. Since we included 14 urological and reproductive health outcomes, the significance threshold was *P* < 0.0036 (0.05/14) after Bonferroni correction. A *P* < 0.05, but above the threshold of Bonferroni correction significance, was considered a suggestive causal association. All the statistical analyses were conducted using R software.

## Results

**Figure 1:**
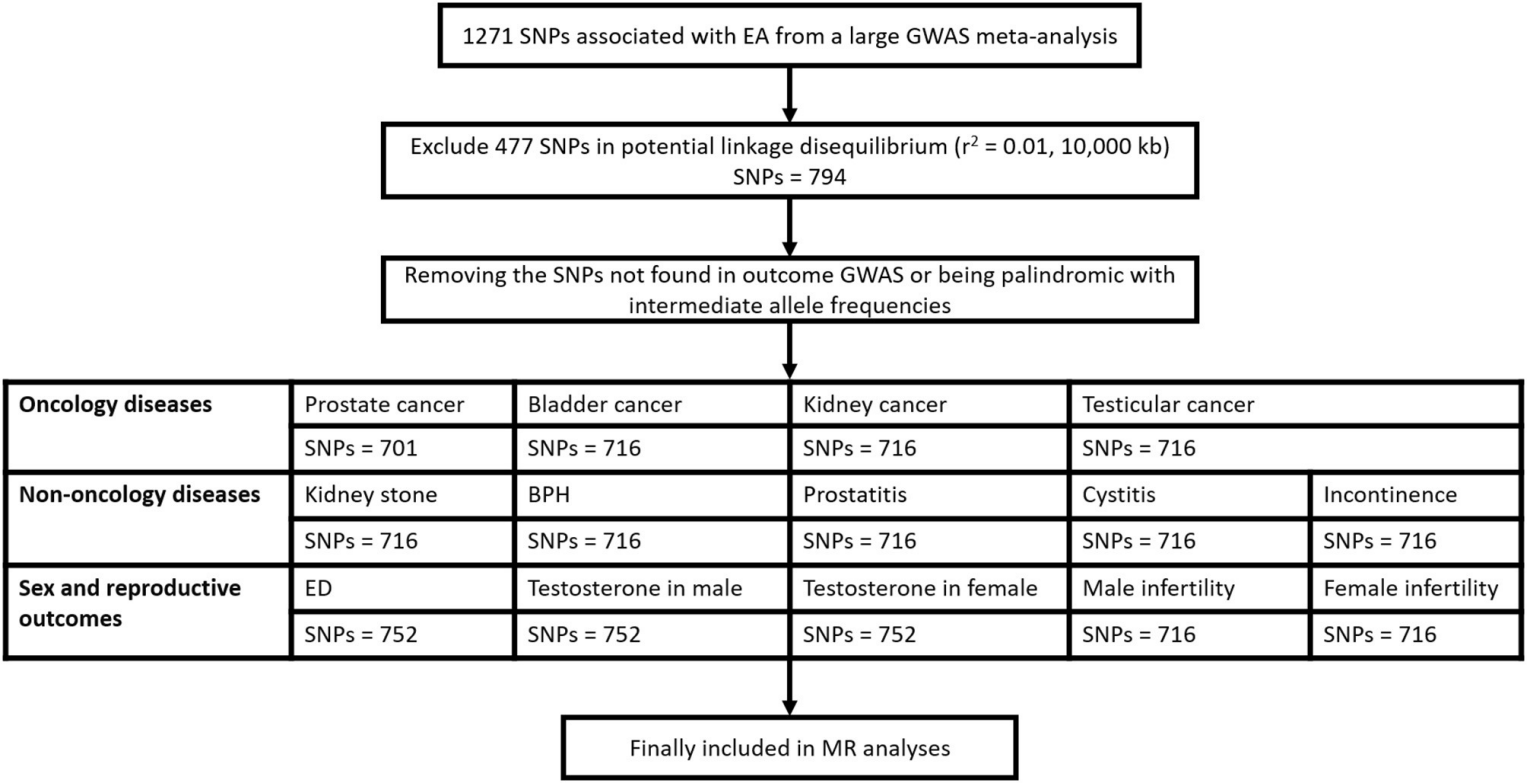
Flow diagram for eligible SNPs selection for each of the 14 outcomes. SNP, single nucleotide polymorphism; EA, educational attainment; GWAS, genome wide association study; BPH, benign prostatic hyperplasia; ED, erectile dysfunction.

A flow diagram for eligible SNPs selection for each of 14 outcomes was presented in Figure 1.

**Figure 2:**
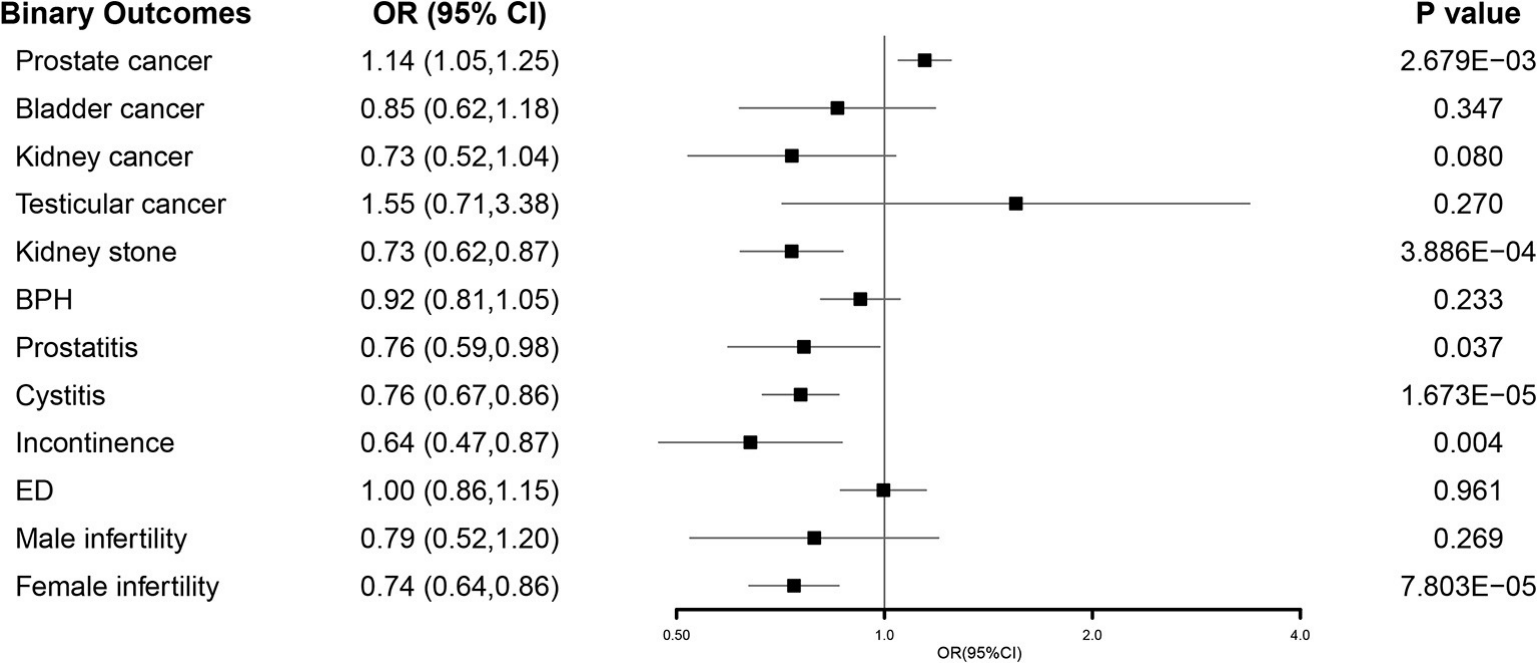
Forest plot of the MR results between EA and 12 binary outcomes including prostate cancer, bladder cancer, kidney cancer, testicular cancer, kidney stone, BPH, prostatitis, cystitis, incontinence, ED, male infertility, and female infertility. MR, Mendelian randomization; EA, educational attainment; BPH, benign prostatic hyperplasia; ED, erectile dysfunction; OR, odds ratio; CI, confidence interval.

For the four oncological diseases, the primary analysis using IVW suggested that genetically predicted one SD increase in EA was causally correlated with a higher risk of prostate cancer [odds ratio (OR) 1.14, 95% confidence interval (CI) 1.05–1.25, *P* = 0.003], while no causal effect was observed for bladder cancer (OR 0.85, 95% CI 0.62–1.18, *P* = 0.347), kidney cancer (OR 0.73, 95% CI 0.52–1.04, *P* = 0.080), and testicular cancer (OR 1.55, 95% CI 0.71–3.38, *P* = 0.270) (Figure 2). However, not all the sensitivity analyses supported the causation between EA and prostate cancer (Supplementary Table 3).

In terms of the five non-oncological diseases, the results from IVW showed that genetically predicted one SD increase in EA was correlated with a decreased risk of kidney stone (OR 0.73, 95% CI 0.62–0.87, *P* < 0.001) and cystitis (OR 0.76, 95% CI 0.67–0.86, *P* < 0.001) after Bonferroni correction and suggestively correlated with a lower risk of prostatitis (OR 0.76, 95% CI 0.59–0.98, *P* = 0.037) and incontinence (OR 0.64, 95% CI 0.47–0.87, *P* = 0.004) (Figure 2). Most of the sensitivity analyses supported the causation between EA and them (Supplementary Table 3). For BPH (OR 0.92, 95% CI 0.81–1.05, *P* = 0.233), no causal relationship was found (Figure 2).

**Figure 3:**
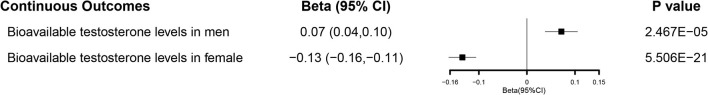
Forest plot of the MR results between EA and two continuous outcomes including bioavailable testosterone levels in male and female. MR, Mendelian randomization; EA, educational attainment; CI, confidence interval.

For the remaining five sexual and reproductive health outcomes, we found that genetically predicted one SD increase in EA was causally associated with a higher testosterone level in men (β 0.07, 95% CI 0.04–0.10, *P* < 0.001) and a lower level (β −0.13, 95% CI−0.16 to−0.11, *P* < 0.001) in women after Bonferroni correction (Figure 3). For infertility, the results from IVW estimates showed that genetically predicted one SD increase in EA was correlated with a lower risk of infertility in females (OR 0.74, 95% CI 0.64–0.86, *P* < 0.001), while no causal effect was observed in males (OR 0.79, 95% CI 0.52–1.20, *P* = 0.269). For ED (OR 1.00, 95% CI 0.86–1.15, *P* = 0.961), no causal relationship was observed (Figure 2). The detailed results of our MR study were presented in Supplementary Table 3.

## Discussion

In the current research, we investigated the causal effects of EA on 14 urological and reproductive health outcomes. Our findings suggested that genetically determined increased EA was correlated with a higher prostate cancer risk and a lower risk of kidney stone, prostatitis, cystitis, and incontinence. For the bioavailable testosterone levels and infertility, sex-specific associations were observed, with genetically determined increased EA being related to higher levels of testosterone in men and lower levels of testosterone in women, and correlated with a decreased risk of infertility in women but was not related to male infertility. In terms of kidney cancer, bladder cancer, testicular cancer, BPH, and ED, no causal effects were observed.

Prostate cancer is a common malignancy worldwide. Prior observational studies have reported that populations with higher EA were at a higher risk of prostate cancer (3, 4), which was in accordance with our finding. Apart from the higher diagnostic activity among the well-educated population (4), another possible explanation for this correlation might be that people with higher EA commonly report higher fat consumption and less physical activity (31), thus increasing the risk of prostate cancer. However, there are few relevant studies, and the underlying mechanism still needs further research.

Kidney stone is another common urological disease. In a prior study, by analyzing the education levels and 24-h urine composition of 435 kidney stone patients, they found that a decreasing level of education was correlated with increased urine calcium, supersaturation of calcium oxalate, and supersaturation of calcium phosphate (9), thus appearing to increase kidney stone formation. However, a significant limitation of this study was that they only enrolled patients with stone formation. Thus, their results might not be generalizable to those without a history of nephrolithiasis. While in our MR analyses, using the GWAS data from 4,969 cases and 213,445 controls, our results were more reliable and generalizable to the general population. In addition to kidney stone incidence risk, EA has also been correlated with the degree of stone burden. In a retrospective study conducted by Bayne (8), after analyzing the socioeconomic and clinical data of 650 patients, they found that a lower education level was correlated with an increased stone burden >2 cm. One possible explanation for the association between EA and kidney stone might be *Oxalobacter formigenes*. Increasing evidence has revealed that *Oxalobacter formigenes* are essential in regulating oxalate homeostasis, with effects that inhibit calcium oxalate stone formation. Researchers found that education level, especially for education levels lower than high school, was associated with an abundance of *Oxalobacter formigenes* after analyzing over 8,000 American Gut Project fecal samples (32).

We also observed that increased EA was correlated with a lower risk of prostatitis and cystitis, which has rarely been reported before. Although the exact underlying mechanism was unknown, one possible explanation was that the population with higher EA are less likely to smoke and more likely to participate in physical activity and have better health habits (33), thus decreasing the risk of prostatitis and cystitis. Regarding incontinence, similar to previous observational studies (12, 13), we found that the population with higher EA were at a lower risk of incontinence. A possible reason could be that individuals with higher EA are inclined to pay more attention to their health and are more willing to take preventive measures to maintain their good health and decrease the risk of incontinence. In contrast, those with lower EA usually perform labor intensive work, which has been regarded as a risk factor for incontinence (34).

In terms of the sexual and reproductive health outcomes, sex-specific associations were observed. We observed that increased EA was causally related to higher levels of testosterone in men and lower levels of testosterone in women, which was partially consistent with prior findings (18). The presence of this association suggested that EA might affect the homeostatic setpoints by which typical hormone concentrations are maintained (18), but the reason for this sex-specific association needs further research. We also observed that increased EA was related to a lower risk of female infertility but was not related to male infertility. Previous studies on the correlation between EA and female infertility have yielded inconsistent results. Two studies (16, 17) reported that EA was inversely associated with female infertility, while one study reported a positive relationship (15). In the current research, we added new evidence to the inverse association between EA and female infertility. This might be because women with higher EA usually have healthier lifestyles and better curative care (16). For male infertility, no causal effect was observed, which was consistent with a prior observational study (15).

For bladder cancer, kidney cancer, and testicular cancer, similar to prior observational studies (5–7), we observed no causality in our study. The role of EA in determining BPH is inconsistent (10, 11), with one study that reported the population with higher EA were at a higher risk (11) and another reporting a lower risk (10). However, all these observational studies are prone to confounding factors, and the results from our MR study indicated that EA might not have a causal effect on BPH. In terms of ED, although a prior observational study reported that increased EA was correlated with a lower risk of ED (14), no causal relationship was observed in the current MR study.

Our study has several strengths. First, as far as we know, this is the first MR study to explore the causal association between EA and urological or reproductive health outcomes. Compared with other observational studies, our research is less vulnerable to confounding factors. Second, all the included individuals within the GWAS were of European-descent, making the potential bias from population stratification minimal. In addition, a total of 14 outcomes were analyzed in our study, which is comprehensive and informative. Nevertheless, our study could not avoid limitations. First, since the large percentage of individuals in the EA exposure GWAS is from the UK Biobank, we extracted GWAS data for 10 outcomes from the FinnGen project to avoid overlap as much as possible, but this also leads to a disadvantage since the FinnGen project is prepublication and the data quality might weaken slightly. However, quality control has already been applied to the FinnGen project, and detailed information can be found on their official site (26). Second, although directional pleiotropy was not detected in our study, heterogeneity was found for part of our results, leading to some potential biases. Third, EA might also correlate with some other factors, such as intelligence, income, testosterone levels (35), which might mediate the effects of EA on the 14 included urological and reproductive health outcomes. However, whether these factors play a mediating role between EA and these 14 outcomes was not included in the primary aim of our study and should be explored in future research.

## Conclusions

Our findings indicated that genetically determined increased EA was correlated with a higher risk of prostate cancer and a lower risk of kidney stone, prostatitis, cystitis, and incontinence. For the bioavailable testosterone levels and infertility, sex-specific associations were observed, with genetically determined increased EA being related to higher levels of testosterone in men, decreased levels of testosterone in women and a lower risk of infertility in women but was not related to male infertility. In terms of kidney cancer, bladder cancer, testicular cancer, BPH, and ED, no causal effects were observed. All of these results indicate that EA plays a vital role in urological diseases, especially in non-oncological outcomes and reproductive health. Further research is needed to examine these findings.

## Data Availability Statement

Publicly available datasets were analyzed in this study and can be accessed via the references we used.

## Ethics Statement

Ethical review and approval was not required for the study on human participants in accordance with the local legislation and institutional requirements. Written informed consent for participation was not required for this study in accordance with the national legislation and the institutional requirements.

## Author Contributions

MW, ZJ, XG, and KW: conceptualization. MW, ZJ, and XG: methodology. CY and XJ: data curation. MW, ZJ, XG, and CY: writing—original draft preparation. XJ, HL, and KW: writing—review and editing. ZJ, HL, and KW: funding acquisition. All authors have read and agreed to the published version of the manuscript.

## Funding

This research was funded by the 1·3·5 Project for Disciplines of Excellence, West China Hospital, Sichuan University (ZYGD18011 and ZYJC18015) and Post-Doctor Research Project, West China Hospital, Sichuan University (2020HXBH016).

## Conflict of Interest

The authors declare that the research was conducted in the absence of any commercial or financial relationships that could be construed as a potential conflict of interest.

## Publisher's Note

All claims expressed in this article are solely those of the authors and do not necessarily represent those of their affiliated organizations, or those of the publisher, the editors and the reviewers. Any product that may be evaluated in this article, or claim that may be made by its manufacturer, is not guaranteed or endorsed by the publisher.
