# Supplementary material for: Causal Associations Between Educational Attainment and 14 Urological and Reproductive Health Outcomes: A Mendelian Randomization Study
Source: Front Public Health. 2021 Oct 28;9:742952. doi: 10.3389/fpubh.2021.742952 (PMC8581244; doi:10.3389/fpubh.2021.742952)

Supplementary Material

**Supplementary Table 1.** STROBE Statement—Checklist of items that should be included in reports of ***case-control studies***

|  | Item No | Recommendation |
| --- | --- | --- |
| **Title and abstract** | 1 | (*a*) Indicate the study’s design with a commonly used term in the title or the abstract  *Mendelian randomization is specified in the title and in the abstract.* |
|  |  | (*b*) Provide in the abstract an informative and balanced summary of what was done and what was found  *Provided in the abstract.* |
| Introduction | | |
| Background/rationale | 2 | Explain the scientific background and rationale for the investigation being reported  *Explained in paragraphs 1-4 of the introduction.* |
| Objectives | 3 | State specific objectives, including any prespecified hypotheses  *Stated in paragraph 5 of introduction.* |
| Methods | | |
| Study design | 4 | Present key elements of study design early in the paper  *Presented in the first paragraph of the Materials and Methods section.* |
| Setting | 5 | Describe the setting, locations, and relevant dates, including periods of recruitment, exposure, follow-up, and data collection  *Available information on EA GWAS is provided in the “Instrumental variables selection” section.*  *Available information on GWAS of 14 outcomes is provided in the “GWAS data sources for 14 urological and reproductive outcomes” section.*  *Supplementary Table 2 provides more details on the outcomes.* |
| Participants | 6 | (*a*) Give the eligibility criteria, and the sources and methods of case ascertainment and control selection. Give the rationale for the choice of cases and controls  *This is described in the “GWAS data sources for 14 urological and reproductive outcomes” section, with reference.* |
|  |  | (*b*) For matched studies, give matching criteria and the number of controls per case  *Does not apply.* |
| Variables | 7 | Clearly define all outcomes, exposures, predictors, potential confounders, and effect modifiers. Give diagnostic criteria, if applicable  *Genetic instruments for EA are described in the “Instrumental variables selection”.*  *Outcomes are described in the “GWAS data sources for 14 urological and reproductive outcome” section and in Supplementary Table 2.* |
| Data sources/ measurement | 8* | For each variable of interest, give sources of data and details of methods of assessment (measurement). Describe comparability of assessment methods if there is more than one group  *Sources of GWASs are provided in the “Instrumental variables selection” and the “GWAS data sources for 14 urological and reproductive outcomes” section, with reference.* |
| Bias | 9 | Describe any efforts to address potential sources of bias  *Directional pleiotropy assessment method is described in the “Statistical analysis” section of the methods.* |
| Study size | 10 | Explain how the study size was arrived at  *This does not directly apply to a Mendelian randomization study, but the choice of genetic instruments was explained in the “Instrumental variables selection” section.* |
| Quantitative variables | 11 | Explain how quantitative variables were handled in the analyses. If applicable, describe which groupings were chosen and why  *Does not apply.* |
| Statistical methods | 12 | (*a*) Describe all statistical methods, including those used to control for confounding  *Mendelian randomization and sensitivity analyses are described in the “Statistical analysis” sections of the methods*. |
|  |  | (*b*) Describe any methods used to examine subgroups and interactions  *Does not apply*. |
|  |  | (*c*) Explain how missing data were addressed  *Does not apply.* |
|  |  | (*d*) If applicable, explain how matching of cases and controls was addressed  *Does not apply.* |
|  |  | (*e*) Describe any sensitivity analyses  *This was done in the “Statistical analysis” section.* |
| Results | | |
| Participants | 13* | (a) Report numbers of individuals at each stage of study—eg numbers potentially eligible, examined for eligibility, confirmed eligible, included in the study, completing follow-up, and analysed  *The number of individuals used for exposures and outcomes were given in the “Instrumental variables selection” and the “GWAS data sources for 14 urological and reproductive outcomes” sections.*  *The number of genetic instruments was explained in the “Instrumental variables selection” section.* |
|  |  | (b) Give reasons for non-participation at each stage  *Does not directly apply to Mendelian randomization studies, but the reasons for excluding genetic variants was provided in the “Instrumental variables selection” section.* |
|  |  | (c) Consider use of a flow diagram  *Flow diagram is presented in the Figure 1.* |
| Descriptive data | 14* | (a) Give characteristics of study participants (eg demographic, clinical, social) and information on exposures and potential confounders  *Available information was given in Supplementary Table 2.* |
|  |  | (b) Indicate number of participants with missing data for each variable of interest  *Does not apply.* |
| Outcome data | 15* | Report numbers in each exposure category, or summary measures of exposure  *This was done in the “GWAS data sources for 14 urological and reproductive outcomes” section and Supplementary Table 2.* |
| Main results | 16 | (*a*) Give unadjusted estimates and, if applicable, confounder-adjusted estimates and their precision (eg, 95% confidence interval). Make clear which confounders were adjusted for and why they were included  *Confounder does not apply to Mendelian randomization, but primary MR analysis is given in the “Results” section, as well as in Figure 2 and 3.* |
|  |  | (*b*) Report category boundaries when continuous variables were categorized  *Does not apply.* |
|  |  | (*c*) If relevant, consider translating estimates of relative risk into absolute risk for a meaningful time period  *Does not apply.* |

*Give information separately for cases and controls.

**Supplementary Table 2.** Detailed description and definition of 14 urological and reproductive health outcomes

| **Oncology diseases** | **N case** | **N control** | **Population** | **Data sources** | **Notes: PMID** | **Definition** |
| --- | --- | --- | --- | --- | --- | --- |
| Prostate cancer | 79,148 | 61,106 | European | PRACTICAL | 29892016 | Clinically or histologically diagnosed prostate cancer |
| Bladder cancer | 1,115 | 174,006 | European | FinnGen | Pre-publication (ID: C3_BLADDER_EXALLC) | ICD-10: C67, ICD-9: 188, ICD-8: 188 |
| Kidney cancer | 971 | 174,006 | European | FinnGen | Pre-publication (ID: C3_KIDNEY_NOTRENALPELVIS_EXALLC) | ICD-10: C64, ICD-9: 1890, ICD-8: 1890 |
| Testicular Cancer | 199 | 74,685 | European | FinnGen | Pre-publication (ID: C3_TESTIS_EXALLC) | ICD-10: C62, ICD-9: 186, ICD-8: 186 |
| **Non-oncology diseases** |  |  |  |  |  |  |
| Kidney stone | 4,969 | 213,445 | European | FinnGen | Pre-publication (ID: N14_CALCUKIDUR) | ICD-10: N20, ICD-9: 592, ICD-8: 592 |
| Hyperplasia of prostate | 13,118 | 72,799 | European | FinnGen | Pre-publication (ID: N14_PROSTHYPERPLA) | ICD-10: N40, ICD-9: 600, ICD-8: 600 |
| Prostatitis | 1,859 | 72,799 | European | FinnGen | Pre-publication (ID: N14_PROSTATITIS) | ICD-10: N41, ICD-9: 601, ICD-8: 601 |
| Cystitis | 8,081 | 195,140 | European | FinnGen | Pre-publication (ID: N14_CYSTITIS) | ICD-10: N30, ICD-9: 595, ICD-8: 595 |
| Incontinence | 1,357 | 202,910 | European | FinnGen | Pre-publication (ID: R18_UNSPE_URINARY_INCONTINENCE) | ICD-10: R32 |
| **Sexual and Reproductive Health** |  |  |  |  |  |  |
| Erectile dysfunction | 6,175 | 217,630 | European | UK biobank | 30583798 | Self-reported or physician-reported erectile dysfunction using ICD10 codes N48.4 and F52.2, or use of oral erectile dysfunction medication (sildenafil/Viagra, tadalafil/Cialis, or vardenafil/Levitra), or a history of surgical intervention for erectile dysfunction (using OPCS-4 codes L97.1 and N32.6) |
| Bioavailable testosterone levels in men | 178,782 | NA | European | UK biobank | 32042192 | Bioavailable testosterone was calculated using the Vermeulen equation |
| Bioavailable testosterone levels in women | 188,507 | NA | European | UK biobank | 32042192 | Bioavailable testosterone was calculated using the Vermeulen equation |
| Male infertility | 680 | 72,799 | European | FinnGen | Pre-publication (ID: N14_MALEINFERT) | ICD-10: N46, ICD-9: 606, ICD-8: 606 |
| Female infertility | 6,481 | 68,969 | European | FinnGen | Pre-publication (ID: N14_FEMALEINFERT) | ICD-10: N97 |

PRACTICAL, Prostate Cancer Association Group to Investigate Cancer-Associated Alterations in the Genome; NA, not available.

**Supplementary Table 3.** Summary on MR results of EA on prostate cancer, bladder cancer, kidney cancer, testicular cancer, kidney stone, BPH, prostatitis, cystitis, incontinence, ED, bioavailable testosterone levels in men, bioavailable testosterone levels in women, male infertility, and female infertility.

| **EXPOSURE** | **OUTCOME** | **MR RESULTS** | | | | | **HETEROGENEITY** | | **PLEIOTROPY** | |
| --- | --- | --- | --- | --- | --- | --- | --- | --- | --- | --- |
|  |  | **Methods** | **N SNPs** | **OR^a^ / β^b^** | **95%CI** | ***P* value** | **METHODS** | ***P* value** | **METHODS** | ***P* value** |
| EA | Prostate cancer | IVW | 701 | 1.14 | 1.05-1.25 | **2.679e-03** | IVW | 1.98e-53 |  |  |
|  |  | MR-Egger |  | 1.07 | 0.78-1.47 | 0.679 | MR-Egger | 1.52e-53 | MR-Egger intercept | 0.67 |
|  |  | Weighted median |  | 1.13 | 1.02-1.25 | **1.669e-02** |  |  |  |  |
|  |  | Weighted mode |  | 1.19 | 0.85-1.65 | 0.312 |  |  |  |  |
|  |  |  |  |  |  |  |  |  |  |  |
| EA | Bladder cancer | IVW | 716 | 0.85 | 0.62-1.18 | 0.347 | IVW | 0.38 |  |  |
|  |  | MR-Egger |  | 1.17 | 0.34-4.04 | 0.803 | MR-Egger | 0.37 | MR-Egger intercept | 0.61 |
|  |  | Weighted median |  | 0.87 | 0.53-1.43 | 0.588 |  |  |  |  |
|  |  | Weighted mode |  | 1.82 | 0.31-10.85 | 0.509 |  |  |  |  |
|  |  |  |  |  |  |  |  |  |  |  |
| EA | Kidney cancer | IVW | 716 | 0.73 | 0.52-1.04 | 0.080 | IVW | 0.60 |  |  |
|  |  | MR-Egger |  | 0.90 | 0.24-3.33 | 0.872 | MR-Egger | 0.59 | MR-Egger intercept | 0.76 |
|  |  | Weighted median |  | 0.64 | 0.38-1.06 | 0.081 |  |  |  |  |
|  |  | Weighted mode |  | 0.36 | 0.05-2.41 | 0.293 |  |  |  |  |
|  |  |  |  |  |  |  |  |  |  |  |
| EA | Testicular cancer | IVW | 716 | 1.55 | 0.71-3.38 | 0.270 | IVW | 0.07 |  |  |
|  |  | MR-Egger |  | 0.55 | 0.03-10.54 | 0.692 | MR-Egger | 0.07 | MR-Egger intercept | 0.48 |
|  |  | Weighted median |  | 1.69 | 0.55-5.20 | 0.357 |  |  |  |  |
|  |  | Weighted mode |  | 0.87 | 0.02-40.35 | 0.945 |  |  |  |  |
|  |  |  |  |  |  |  |  |  |  |  |
| EA | Kidney stone | IVW | 716 | 0.73 | 0.62-0.87 | **3.886e-04** | IVW | 7.49e-04 |  |  |
|  |  | MR-Egger |  | 0.43 | 0.22-0.82 | **1.036e-02** | MR-Egger | 9.02e-04 | MR-Egger intercept | 0.09 |
|  |  | Weighted median |  | 0.68 | 0.53-0.87 | **2.240e-03** |  |  |  |  |
|  |  | Weighted mode |  | 0.60 | 0.25-1.45 | 0.260 |  |  |  |  |
|  |  |  |  |  |  |  |  |  |  |  |
| EA | BPH | IVW | 716 | 0.92 | 0.81-1.05 | 0.233 | IVW | 1.98e-05 |  |  |
|  |  | MR-Egger |  | 0.81 | 0.49-1.33 | 0.402 | MR-Egger | 1.84e-05 | MR-Egger intercept | 0.59 |
|  |  | Weighted median |  | 0.92 | 0.77-1.10 | 0.357 |  |  |  |  |
|  |  | Weighted mode |  | 0.94 | 0.48-1.82 | 0.850 |  |  |  |  |
|  |  |  |  |  |  |  |  |  |  |  |
| EA | Prostatitis | IVW | 716 | 0.76 | 0.59-0.98 | **0.037** | IVW | 0.47 |  |  |
|  |  | MR-Egger |  | 0.63 | 0.24-1.63 | 0.338 | MR-Egger | 0.46 | MR-Egger intercept | 0.67 |
|  |  | Weighted median |  | 0.73 | 0.50-1.08 | 0.113 |  |  |  |  |
|  |  | Weighted mode |  | 0.68 | 0.20-2.31 | 0.534 |  |  |  |  |
|  |  |  |  |  |  |  |  |  |  |  |
| EA | Cystitis | IVW | 716 | 0.76 | 0.67-0.86 | **1.673e-05** | IVW | 0.096 |  |  |
|  |  | MR-Egger |  | 0.62 | 0.38-1.01 | 5.308e-02 | MR-Egger | 0.095 | MR-Egger intercept | 0.41 |
|  |  | Weighted median |  | 0.68 | 0.56-0.81 | **4.304e-05** |  |  |  |  |
|  |  | Weighted mode |  | 0.55 | 0.28-1.10 | 9.103e-02 |  |  |  |  |
|  |  |  |  |  |  |  |  |  |  |  |
| EA | Incontinence | IVW | 716 | 0.64 | 0.47-0.87 | **0.004** | IVW | 4.1e-02 |  |  |
|  |  | MR-Egger |  | 0.39 | 0.12-1.24 | 0.112 | MR-Egger | 4.0e-02 | MR-Egger intercept | 0.39 |
|  |  | Weighted median |  | 0.64 | 0.42-0.99 | **0.045** |  |  |  |  |
|  |  | Weighted mode |  | 1.76 | 0.35-8.78 | 0.490 |  |  |  |  |
|  |  |  |  |  |  |  |  |  |  |  |
| EA | ED | IVW | 752 | 1.00 | 0.86-1.15 | 0.961 | IVW | 0.26 |  |  |
|  |  | MR-Egger |  | 1.35 | 0.80-2.27 | 0.265 | MR-Egger | 0.27 | MR-Egger intercept | 0.24 |
|  |  | Weighted median |  | 1.21 | 0.97-1.50 | 0.088 |  |  |  |  |
|  |  | Weighted mode |  | 1.57 | 0.81-3.03 | 0.178 |  |  |  |  |
|  |  |  |  |  |  |  |  |  |  |  |
| EA | Bioavailable testosterone levels in men | IVW | 752 | 0.07 | 0.04-0.10 | **2.467E-05** | IVW | 1.14e-79 |  |  |
|  |  | MR-Egger |  | 0.14 | 0.02-0.26 | **2.243E-02** | MR-Egger | 1.86e-79 | MR-Egger intercept | 0.24 |
|  |  | Weighted median |  | 0.07 | 0.03-0.10 | **2.114E-04** |  |  |  |  |
|  |  | Weighted mode |  | 0.13 | -0.01-0.28 | 7.761E-02 |  |  |  |  |
|  |  |  |  |  |  |  |  |  |  |  |
| EA | Bioavailable testosterone levels in female | IVW | 752 | -0.13 | -0.16- -0.11 | **5.506E-21** | IVW | 1.89e-84 |  |  |
|  |  | MR-Egger |  | -0.12 | -0.22- -0.02 | **1.915E-02** | MR-Egger | 1.27e-84 | MR-Egger intercept | 0.81 |
|  |  | Weighted median |  | -0.11 | -014- -0.08 | **5.770E-14** |  |  |  |  |
|  |  | Weighted mode |  | -0.07 | -0.21-0.07 | 0.350 |  |  |  |  |
|  |  |  |  |  |  |  |  |  |  |  |
| EA | Male infertility | IVW | 716 | 0.79 | 0.52-1.20 | 0.269 | IVW | 0.36 |  |  |
|  |  | MR-Egger |  | 1.86 | 0.39-8.97 | 0.439 | MR-Egger | 0.36 | MR-Egger intercept | 0.27 |
|  |  | Weighted median |  | 0.87 | 0.46-1.67 | 0.685 |  |  |  |  |
|  |  | Weighted mode |  | 0.96 | 0.11-8.48 | 0.973 |  |  |  |  |
|  |  |  |  |  |  |  |  |  |  |  |
| EA | Female infertility | IVW | 716 | 0.74 | 0.64-0.86 | **7.803e-05** | IVW | 4.9e-03 |  |  |
|  |  | MR-Egger |  | 0.76 | 0.43-1.34 | 3.424e-01 | MR-Egger | 4.5e-03 | MR-Egger intercept | 0.92 |
|  |  | Weighted median |  | 0.74 | 0.60-0.92 | **7.289e-03** |  |  |  |  |
|  |  | Weighted mode |  | 0.47 | 0.21-1.05 | 6.624e-02 |  |  |  |  |

^a^ For binary outcomes

^b^ For continuous outcomes

MR, Mendelian randomization; EA, educational attainment; BPH, benign prostatic hyperplasia; ED, erectile dysfunction; SNP, single nucleotide polymorphism; OR, odds ratio; IVW, inverse variance weighted

**Supplementary Figure 1.** Study design of the current Mendelian randomization research


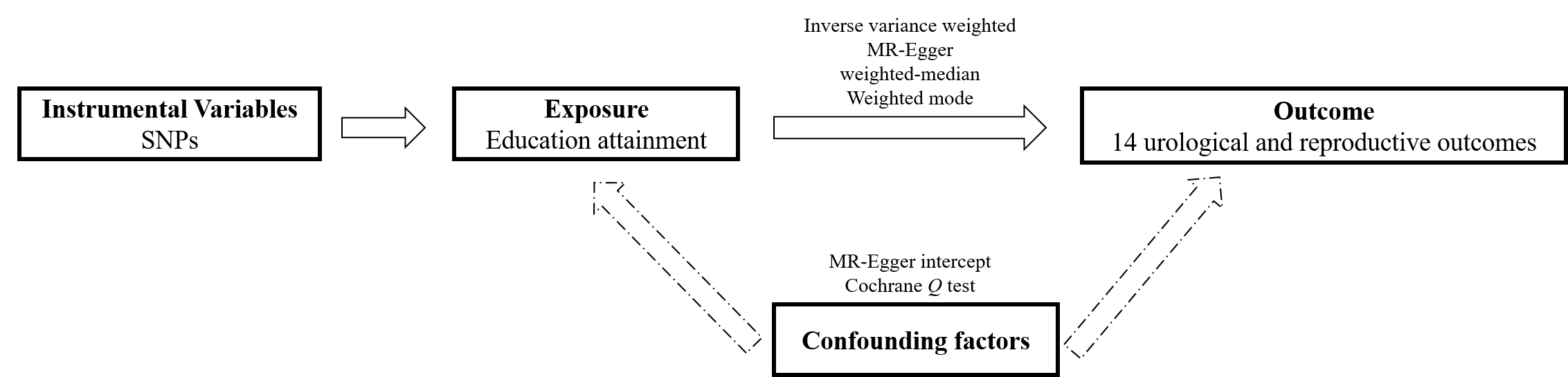

Supplement: Supplementary file 1 [file Table_1.DOCX]
